# Symptoms and Side Effects of Bacille Calmette–Guerin Therapy for Non-Muscle Invasive Bladder Cancer as Reported by Patients: A Systematic Review

**DOI:** 10.3390/cancers17020160

**Published:** 2025-01-07

**Authors:** John Lahoud, Alfin Okullo, Claudia Rutherford, David P. Smith, Daniel S. J. Costa, Margaret-Ann Tait, Shomik Sengupta, Manish I. Patel

**Affiliations:** 1 Department of Urology, Westmead Hospital, Sydney, NSW 2145, Australia; jlah4911@uni.sydney.edu.au (J.L.);; 2 Specialty of Surgery, Sydney Medical School, University of Sydney, Sydney, NSW 2050, Australia; 3 School of Psychology, University of Sydney, Sydney, NSW 2050, Australia; 4 Cancer Nursing Research Unit, Sydney Nursing School, University of Sydney, Sydney, NSW 2050, Australia; 5 Cancer Research Division, Cancer Council New South Wales, Sydney, NSW 2011, Australia; 6 Sydney School of Public Health, University of Sydney, Sydney, NSW 2050, Australia; 7 Menzies Health Institute Queensland, Griffith University, Southport, QLD 4222, Australia; 8 School of Public Health and Preventative Medicine, Monash University, Clayton, VIC 3168, Australia; 9 Eastern Health Clinical School, Monash University, Box Hill, Melbourne, VIC 3800, Australia; shomik.sengupta@monash.edu; 10 Department of Urology, Eastern Health, Box Hill, VIC 3128, Australia; 11 Australian and New Zealand Urogenital and Prostate (ANZUP) Cancer Trials Group, Barangaroo, Sydney, NSW 2000, Australia

**Keywords:** non-muscle invasive bladder cancer, chemotherapy, side effects, symptoms of treatment, patient reported outcomes

## Abstract

Background/Objectives: Knowledge of the symptoms and side effects (SSEs) of Bacille Calmette–Guerin (BCG) therapy for non-muscle invasive bladder cancer (NMIBC) is critical when establishing selecting appropriate therapies for patients. The aim of our study was to systematically review the common patient-reported SSEs associated with BCG-based and other intravesical chemotherapy treatment options for NMIBC. Methods: A systematic search of AMED, MEDLINE, EMBASE, PsycINFO, Web of Knowledge, and Scopus was conducted from inception to July 2024. The PRISMA process was followed. Prospective studies with an adult cohort that assessed SSEs through direct patient reports with standardized patient-reported outcome measures were included in this study. A narrative synthesis was performed to compare the frequency of SSEs reported by treatment options. Statistical analysis was performed using chi square and Fisher’s exact tests, with statistical significance at *p* < 0.05. Results: Thirty-four studies met the eligibility criteria. The main findings indicated that BCG induction is more toxic than BCG induction with maintenance; however, severe SSEs resulting in treatment cessation occurred almost twice as often in patients on BCG induction with maintenance. Patients who received full-dose BCG were more likely to have SSEs compared to those receiving a low dose. BCG monotherapy alone caused more SSEs compared to BCG with chemotherapy. Patients reported more SSEs with BCG compared to chemotherapy alone for induction with maintenance. Limitations of the study include the varied length of maintenance regimes affecting nature of data reported. Conclusions: The findings of this study allow for improved counselling of patients regarding expected side effects in accordance with their recommended treatment options for NMIBC.

## Simple Summary

We explored the symptoms and side effects that can result from using BCG (an immunotherapy agent) to treat bladder cancer. We concluded that when BCG was combined with chemotherapy and at lower doses, the symptoms and side effects were less reported. These findings allow clinicians to improve counselling of patients regarding the management course and side effects for treating NMIBC.

## 1. Introduction

Non-muscle invasive bladder cancer (NMIBC) involves tumor invasion up to the lamina propria, comprising approximately 75% of all newly diagnosed bladder cancers [1]. NMIBC can have a high recurrence rate and be associated with increased financial expenditure for the public health system [2]. The American Urological Association and European Association of Urology guidelines recommend additional therapy following transurethral resection of bladder tumor for NMIBC for all risk groups, to reduce the risk of recurrence [3,4]. These clinical practice recommendations are almost exclusively based on oncological outcomes, with shortcomings on focus regarding treatment toxicity [5].

The European Association of Urology (EAU) and American Urological Association (AUA) have provided guidelines for the management of NMIBC and suggestions for induction and maintenance schedules of chemotherapy and immunotherapy agents [6,7]. As per the EAU guidelines, a complete Bacillus Calmette–Guerin (BCG) schedule involves a 6-weekly induction phase of instillations, followed by maintenance phase of 3 weekly instillations at 3, 6, 12, 18, 24, 30 and 36 months [6]. Both the EAU and AUA support maintenance BCG for three years in patients with high-risk disease, as compared to patients with lower or intermediate risk tumors that require one-year maintenance therapy for optimal efficacy of treatment [6,7]. Furthermore, the AUA suggests in patients with suspected or known low or intermediate risk bladder cancer that a single postoperative instillation of intravesical chemotherapy such as gemcitabine or mitomycin C is advisable within 24 h of TURBT [7]. In patients who have a suspected perforation or extensive resection, intravesical chemotherapy is not recommended [6,7].

Intravesical BCG is used as first-line adjuvant therapy for intermediate and high-risk NMIBC [8]. Depending on the risk group of NMIBC, a protocol for BCG maintenance is recommended in addition to BCG induction [3]. While it has been considered safe to use, studies have demonstrated more than 70% of patients experience side effects and approximately 8% cease treatment due to toxicity [8,9]. Furthermore, recent logistical issues with BCG have led to a worldwide BCG shortage; thus, the need for ongoing research and interest in alternatives including chemotherapies such as mitomycin C, gemcitabine, and others [8,10].

BCG failures are generally described as any high-grade disease that occurs during or after BCG therapy. Per the EAU guidelines, for patients with BCG-unresponsive disease—that is, the development of T1/Ta high grade recurrence within 6 months of adequate BCG exposure (or carcinoma in situ within 12 months)—further BCG is unlikely to be effective, and thus radical cystectomy is the preferred option [6]. The AUA guidelines also recommend, in patients who are unwilling or unfit for cystectomy clinical trial enrolment, alternative intravesical chemotherapies, or systemic immunotherapy in patients with CIS [7].

Adjuvant intravesical therapies can cause significant local and systemic side effects, thus, affecting adherence to treatment and patient-reported outcomes (PROs) [11]. For instance, one trial demonstrated only a 16% completion rate of full treatment in patients who underwent BCG induction with maintenance due to treatment toxicity [12,13]. Previously published systematic reviews of intravesical therapies for NMIBC management have not reported on side effects or PROs according to treatment type. Furthermore, there have been some discrepancies between studies regarding toxicity between full and low-dose BCG therapy. In a study by the Spanish Oncology Group (CUETO), patients who had reduced-dose BCG had significantly less treatment toxicity; however, Oddens et al. found no differences in treatment toxicity between the two in induction and maintenance regimens [14,15]. Therefore, the aim of our study was to systematically review the common patient-reported SSEs associated with BCG via different regimens and compare the frequency of symptoms reported by treatment options and regimens.

## 2. Materials and Methods

### 2.1. Eligibility Criteria

Studies were eligible for inclusion if they were primary research of prospective quantitative design (i.e., randomized controlled trials (RCT), a cohort study or comparative study), included adult patients (over 18 years of age) with any grade of NMIBC from any setting, primary or secondary outcomes assessed were symptoms, side effects and/or toxicities through direct patient reports, and had sufficient data on PROs reported that allowed for extraction of symptom results by treatment. Studies were excluded if the sample included patients with stage ≥ T2 muscle-invasive bladder cancer, symptoms were assessed by a healthcare provider or proxy, limited to pediatric populations, a study was a retrospective review of medical records, qualitative or a conference abstract, or the study population included re-treatment cases. Searches were limited to papers published in the English language. This systematic review is not registered in any database.

### 2.2. Information Sources

A systematic search of AMED, MEDLINE, EMBASE, PsycINFO, Web of Knowledge, and Scopus from inception to July 2024 was conducted.

### 2.3. Search Strategy

The search strategy comprised terms for NMIBC and treatment symptoms and side effects. We also searched the reference lists of included studies and relevant systematic reviews, and names of selected key authors identified in relevant literature.

### 2.4. Selection Process

**Figure 1:**
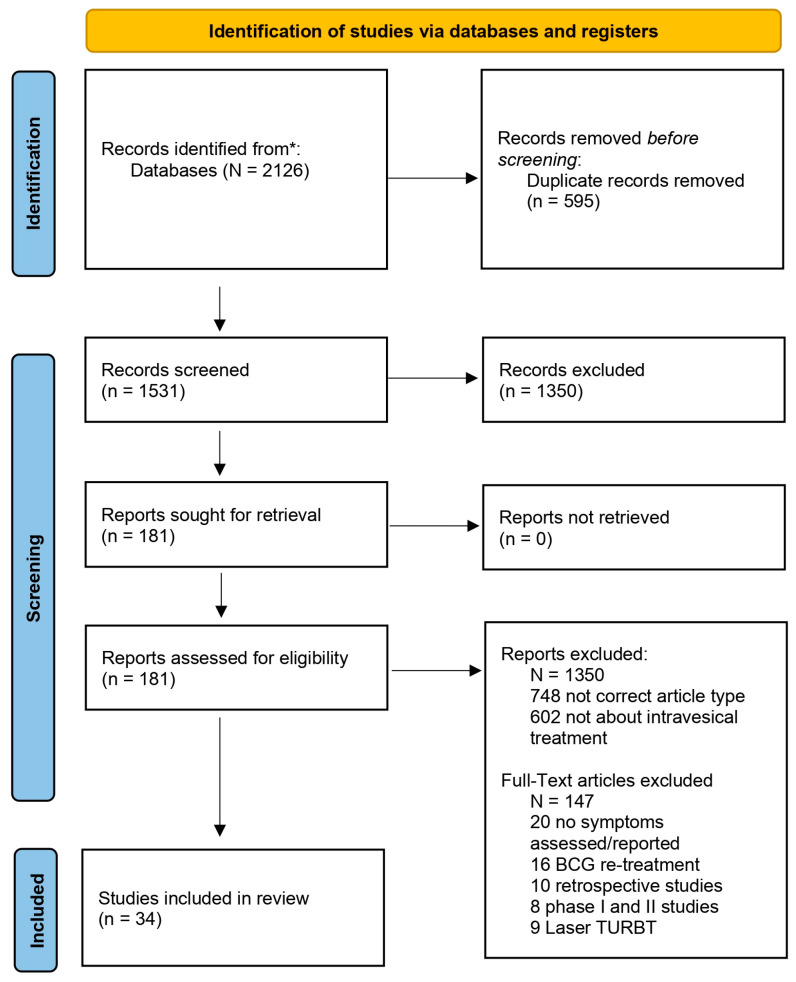
Preference Reporting Items for Systematic Reviews and Meta-Analyses flow chart. * refers to databases in Section 2.2 Information Sources.

The Preferred Reporting Items for Systematic Reviews and Meta-analyses (PRISMA) process was followed (Figure 1). Retrieved titles and abstracts were initially screened against the eligibility criteria by one reviewer (A.O., C.R., or M.A.T.). Studies that did not have the correct article type, treatment schedules, did not report any symptoms, and studies that were retrospective phase I and II studies were excluded. Two reviewers (J.L. and A.O.) independently assessed full text papers against the eligibility criteria, with final inclusion confirmed in consultation with a more senior co-author (C.R.). In case of disagreement, mediation by a senior author (M.I.P.) was sought (Figure 1).

### 2.5. Data Collection Process

Study aims, sample characteristics, design and methods, and treatment-related SSE results were extracted for each included study and tabulated. Data were extracted by two reviewers (J.L. and A.O.) and queries were discussed and resolved with senior investigators (C.R. and M.I.P.). Specifically, terms used to describe SSEs were extracted, and the number of patients reporting each SSE across all included studies was determined.

### 2.6. Study Risk of Bias Assessment

Given that our review aimed to synthesize treatment-related SSEs, individual quality components of the study methodology were not used as a threshold for the inclusion of primary studies. We included all available data and assessed the appropriateness of each study by making a judgement about whether a study used appropriate methods to address our review questions and to ensure that findings about any treatment SSEs were reported by patients. To ensure data quality and to reduce the risk of bias in the source PRO data, we included only prospective studies that collected treatment-related SSEs using a standardized method across all patients and reported results allowing the percentage of the sample experiencing each symptom to be determined.

### 2.7. Synthesis Methods

Since many studies pooled multiple symptoms rather than reported results for single symptoms, we further amalgamated these symptoms into symptom groups where we determined that despite the difference in terminology, the authors were more than likely describing the same symptom. Symptom frequency was determined for the following symptom or symptom groups: irritative lower urinary tract symptoms (LUTS) (including frequency, pollakisuria, nocturia), dysuria/stranguria/bladder cramps/pain, hematuria, obstructive symptoms, cystitis (chemical or unknown), fever, fatigue/malaise, prostatitis/epididymitis, arthralgia/hepatitis/rash/allergic reaction, BCG sepsis, severe local bladder complications (contraction, ulcerating cystitis), and severe side-effects requiring treatment cessation. To ensure that the reported symptoms were more than likely associated with a particular therapy, therapies were classified into pure therapy groups so that patients only contributed results for one therapy or combination therapy as specified. Statistical analysis was performed using the chi square test and Fisher’s exact test, with statistical significance considered at *p* < 0.05.

## 3. Results

### 3.1. Study Selection

We retrieved 2126 records, of which 34 met the eligibility criteria, with a total of 7070 patients (Table S1).

#### Study Characteristics

Twenty-one studies were randomized prospective trials, and thirteen studies were prospective cohort studies. Sixteen studies were conducted in Europe, eleven in Asia, three in Africa, two in the Middle East, and two in North America.

### 3.2. Results of Syntheses

#### 3.2.1. BCG Induction Versus BCG Induction with Maintenance

**Figure 2:**
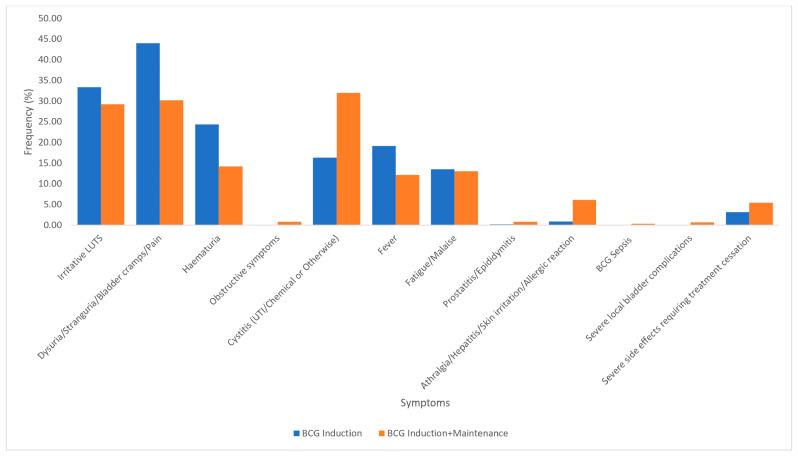
Symptoms and side effects associated with BCG induction only versus BCG induction with maintenance.

A total of 1311 patients had BCG induction only weekly for six to eight weeks (Figure 2). In one study, 30 patients were administered BCG for 12 weeks [16]. Most studies reported treatment toxicity post-instillation and/or at various follow-up intervals post-therapy. A total of 3251 patients had BCG induction with maintenance (Figure 2). For patients who had BCG induction only, the following symptoms occurred more frequently compared to patients who had BCG induction with maintenance: LUTS (33.3% vs. 29.2%, *p* = 0.006), bladder pain (44.0% vs. 30.2%, *p* ≤ 0.001), hematuria (24.3% vs. 14.2%, *p* ≤ 0.001), and fever (19.2% vs. 12.1%, *p* ≤ 0.001) (Figure 2). Cystitis (32.0% vs. 16.3%, *p* ≤ 0.001), severe local bladder complications (0.7% vs. 0%, *p* = 0.001), BCG sepsis (0.3% vs. 0.1%, *p* = 0.195), prostatitis/epididymitis (0.8% vs. 0.2%, *p* = 0.007). Severe SEEs requiring treatment cessation (3.1% vs. 5.4%, *p* ≤ 0.001) were less prevalent in those who had BCG induction alone (Figure 2).

#### 3.2.2. BCG Full Dose Versus BCG Low Dose

**Figure 3:**
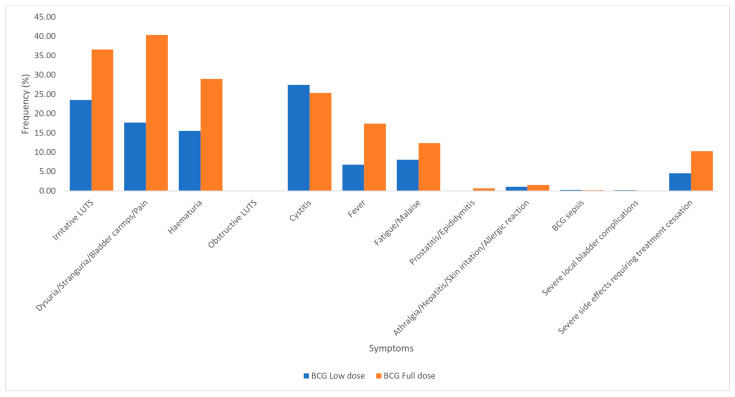
Symptoms and side effects associated with BCG full dose versus BCG low dose.

A total of 4499 patients received BCG, with 2981 receiving a full dose (at least 75 mg of BCG or 2 × 10^6^ CFU) and 1518 receiving low-dose BCG (any dose less than 75 mg of BCG or 2 × 10^6^ CFU) (Figure 3). SSEs were more frequent among patients who received full-dose BCG compared to low-dose BCG as follows: irritative LUTS (36.6% vs. 23.5%, *p* ≤ 0.001), bladder pain (40.4% vs. 17.6%, *p* ≤ 0.001), hematuria (29.0% vs. 15.5%, *p* ≤ 0.001), fever (17.4% vs. 6.8%, *p* ≤ 0.001), fatigue/malaise (12.3% vs. 8.0%, *p* ≤ 0.001), prostatitis/epididymitis (0.7% vs. 0.1%, *p* = 0.002), and severe SSEs requiring treatment cessation (10.2% vs. 4.5%, *p* ≤ 0.001) (Figure 3).

#### 3.2.3. Induction Regimens: BCG Monotherapy as Induction Versus BCG with Sequential Chemotherapy as Induction

**Figure 4:**
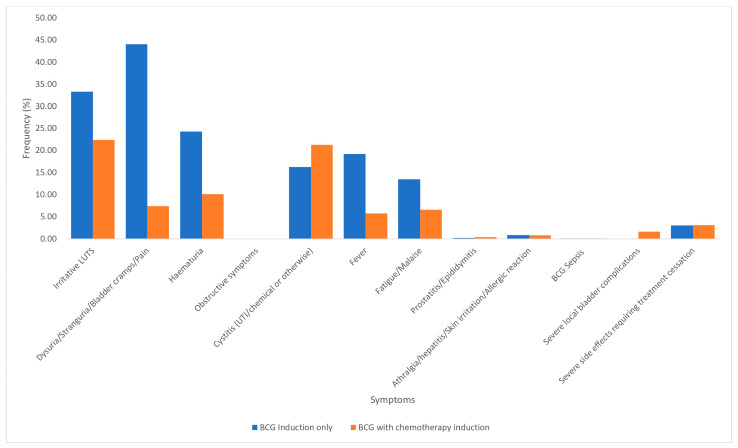
Symptoms and side effects associated with induction: BCG monotherapy versus BCG with chemotherapy.

For induction, 1311 patients received BCG monotherapy, whereas 729 patients received BCG sequentially with chemotherapy (Figure 4). Of the latter, 197 had BCG with mitomycin and 532 had BCG with epirubicin. The following symptoms were more frequent among patients who received BCG monotherapy: irritative LUTS (33.3% vs. 22.4%, *p* ≤ 0.001), bladder pain (44.0% vs. 7.4%, *p* ≤ 0.001), hematuria (24.3% vs. 10.2%, *p* ≤ 0.001), and fever (19.2% vs. 6.6%, *p* ≤ 0.001) (Figure 4). Cystitis (21.3% vs. 16.3%, *p* = 0.007) and severe local bladder complications (0% vs. 1.7%, *p* ≤ 0.001) were less common in patients receiving BCG and chemotherapy (Figure 4). Treatment cessation rates were similar (3.2% vs. 3.1%, *p* = 0.894) (Figure 4).

#### 3.2.4. Induction with Maintenance Regimens: BCG Induction with Maintenance Versus Chemotherapy Induction with Maintenance

**Figure 5:**
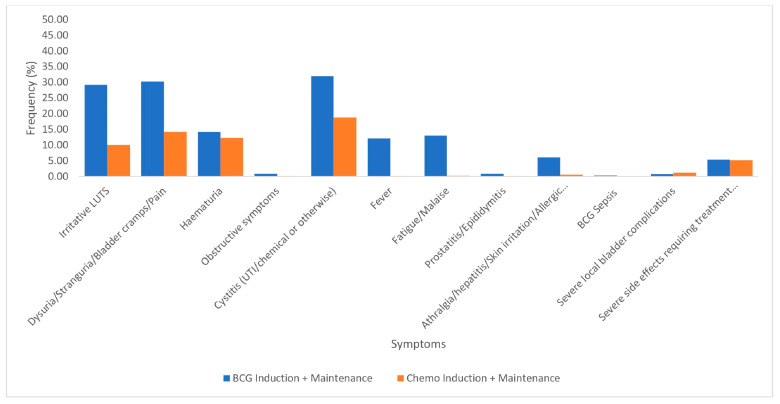
Symptoms and side effects associated with induction and maintenance: BCG versus chemotherapy.

A total of 3251 patients received BCG induction with maintenance, whereas 2451 patients received intravesical chemotherapy induction with maintenance (Figure 5). Chemotherapy induction with maintenance regimens included epirubicin (1578), mitomycin (508), doxorubicin (134), pirarubicin (60), hydroxycamptothecin (61), thiotepa (56), and gemcitabine (54). BCG as compared to chemotherapy was more toxic as follows: irritative LUTS (29.2% vs. 10.0%, *p* ≤ 0.001), bladder pain (30.2% vs. 14.1%, *p* ≤ 0.001), hematuria (14.2% vs. 12.4%, *p* = 0.054), cystitis (32.0% vs. 18.8%, *p* ≤ 0.001), and fever (12.1% vs. 0.2%, *p* ≤ 0.001) (Figure 5). Severe local bladder complications (0.7% vs. 1.1%, *p* = 0.72) were more common among patients receiving chemotherapy. This was not statistically significant. Treatment cessation due to SSEs was comparable (5.4% vs. 5.2%, *p* = 0.952) (Figure 5).

#### 3.2.5. Certainty of Evidence

All SSEs were able to be extracted from the included studies and reported on in this systematic review. Whilst all studies included prospective trials, the heterogenous nature of the data gives our study a moderate certainty of evidence. 

## 4. Discussion

To our knowledge, this is the first review to report patient-reported SSEs for patients who had undergone BCG and other intravesical therapies for NMIBC. The principal findings of this study indicate that BCG induction alone was more toxic than BCG induction with maintenance, that BCG in combination with intravesical chemotherapy was less toxic than BCG monotherapy, that full-dose BCG was more toxic than low-dose BCG, and finally, BCG induction with maintenance was more toxic than chemotherapy with induction and maintenance.

PRO measures have become an imperative component in patient care and clinician understanding of the patient’s journey. Evaluating PROs for bladder cancer has become increasingly prevalent in the current literature to assess symptoms and quality of life [17,18]. For PROs to be effective, they need to successfully capture the patient perspective and priorities relating to the health condition [19]. Furthermore, to best capture these, it is preferable to use rigorously developed and psychometrically sound PRO measures as opposed to study-specific measures to ensure the reliability of data collected [20,21]

Initially, few cancer trials and research have used PROs, as they were previously labelled as subjective measures; however, PROs have been shown to provide invaluable evidence of the impact of current and new treatments on patient symptoms, assisting physicians in providing customized care for patients, empowering patient self-management, and providing a value-based care assessment that assists health care decision-makers in public reporting and policy [22].

Our review suggests that BCG induction is more toxic than BCG induction with maintenance (Figure 2). This is not an isolated observation, as previous studies have found that SSEs were more common during induction and reduced during maintenance therapy [11,12,13,23,24]. Brausi et al. in 2014 reported similar findings when comparing low-dose and full-dose BCG with 1- and 3-year maintenance regimens, observing that most side effects occurred during the first year of treatment, suggesting that SSEs depended on a host factors, rather than on the number of instillations [9]. Their findings were also corroborated in a prior study by van der Meijden et al. 2003, who found less toxicity during BCG maintenance therapy [25]. Furthermore, a recent systematic review by Rutherford et al. 2021 found that PROs were no worse during maintenance when compared to induction, except possibly in role (social and occupational) and cognitive function domains, and nausea and appetite loss symptoms [20]. Contrary to the above findings, a prospective randomized study of 68 patients with high-risk NMIBC that compared maintenance versus single course of intravesical BCG found that overall local adverse events were significantly higher in patients who underwent a maintenance treatment protocol [26].

Severe SSEs resulting in treatment cessation occurred almost twice as frequently in patients on BCG induction with maintenance as compared to those who had BCG induction only (*p* < 0.05) (Figure 2). Brausi et al. found that almost twice as many patients ceased treatment with BCG during the first year (5.4% of patients) compared to the second or third year of treatment (3.2% of patients), suggesting that the longer the treatment period, the lower the likelihood of treatment cessation due to side effects. It is important to consider however, that there are pitfalls associated in using treatment cessation as an endpoint as patients who do not tolerate treatment may drop out of studies and therefore cease PRO assessment despite contributing to the final analysis [27]. This may lead to a misrepresentation of the proportion of SSEs experienced in patients, as the SSE experienced by patients with discontinued treatment may not be accounted for, despite being included in the final analysis. For instance, in the final results of the EORTC-GU Cancers Group randomized study of BCG in intermediate and high-risk Ta, T1 papillary carcinoma of the bladder, 7.8% of patients ceased treatment due to local or systemic adverse events; however, in the randomized study by Lamm et al. (2000), only 16% of patients received all eight scheduled maintenance courses during three years [5,15]. Therefore, our findings suggest that patients should be counselled on the evidence-based response and SSEs of BCG treatment, with encouragement to persist through the induction phase and first year of maintenance, as this could improve compliance [28].

Our results indicated that patients who received full-dose BCG were more likely to have SSEs compared to those who received low-dose BCG (Figure 3). Due to BCG toxicity, attempts at dose reduction without compromising efficacy have been investigated. A randomized controlled study by Oddens et al. in 2013 found that a third dose of BCG with maintenance at one year was suboptimal when compared to a standard dose maintenance at three years and found no differences in toxicity [15]. The Club Urologica Espanol de Tretamiento Oncologico (CUETO) published long-term follow-up outcomes of a randomized study comparing the efficacy and toxicity of standard-dose BCG compared with one third dose BCG. They found that the one-third dose BCG had similar results for recurrence and progression as a full-dose BCG, with significantly less toxicity [14]. However, patients with multifocal tumors managed better with the standard dose, with a trend towards better recurrence rates in patients with high-risk tumors [14]. Furthermore, a network meta-analysis of 24 studies by Kawada et al. in 2023 found that standard-dose BCG was associated with higher risk of adverse events [29]. While there is currently insufficient evidence to support the non-inferiority of a reduced dose BCG administration, the current evidence is in keeping with our findings, suggesting that low-dose BCG is less toxic.

For induction regimens, we found that BCG alone caused more SSEs compared to BCG monotherapy with chemotherapy combination (Figure 4). This finding is supported by the meta-analysis by Huang et al. in 2019, who found that combining BCG with chemotherapy appeared to be an effective treatment for patients with intermediate-to-high risk NMIBC, with a statistically significant better overall survival rate, recurrence-free survival, and disease-specific survival compared to BCG therapy alone [30]. Furthermore, the authors demonstrated that treatment toxicity was less common among patients on combination therapy [30]. In their randomized controlled phase II trial, Di Stasi et al. in 2006 demonstrated that BCG with mitomycin was less toxic than BCG monotherapy for induction and one-year maintenance regimen in patients with pT1 NMIBC, demonstrating higher disease-free interval, lower recurrence and progression rates, and improved disease-specific and overall survival [31].

Finally, our results indicated that patients reported more SSEs with BCG compared to chemotherapy alone for induction with maintenance (Figure 5). Similar to our findings, Tabayoyong et al. in their systematic review of 16 RCTs in 2018 found that treatment toxicity was mild with very little treatment cessation in patients who had epirubicin, MMC, and doxorubicin [32]. The most common side effects were cystitis, dysuria, and hematuria [32]. Additionally, a prospective observational study in 2023 of 183 patients found that intermediate to high-risk NMIBC patients who had BCG induction and maintenance had a higher percentage of grade 1 to 3 adverse events compared to those who had intravesical MMC [33]. These findings may be explained by the fact that BCG administration induces the production of inflammatory cytokines, thus leading to prominent local and systemic side effects, whereas chemotherapies are not normally systemically absorbed in large amounts, acting through a direct cytotoxic effect, with local side effects only [34,35]. Therefore, the implication of these results is that in frail and elderly patients with pre-existing significant LUTS and intermediate-risk NMIBC, intravesical chemotherapy may be a reasonable alternative, particularly when considering BCG shortages.

Our study was limited by the heterogenous studies and thus data reported. Several included studies pooled patient-reported symptoms for maintenance schedules that differed in length, precluding more thorough determination of effects according to the duration of the therapy, and thus, differences in timing of SSEs evaluation. Another limitation is that only an incidence of SSEs was evaluated in this study

The current literature and published systematic reviews on intravesical therapy for NMIBC have largely focused on oncological outcomes, with little attention to PRO data to determine the toxicity associated with these therapies [36,37]. Furthermore, the availability of data on treatment toxicity is frequently in summary form, and not necessarily based on PROs, despite evidence demonstrating their benefit in patient care [38,39,40]. Our study aimed to address these knowledge gaps by presenting PRO data with a breakdown of SSEs according to treatment type and duration.

## 5. Conclusions

Future studies should assess PROs in addition to oncological outcomes associated with treatment for NMIBC. Our systematic review has demonstrated that BCG monotherapy is associated with the highest frequency of SSEs. When BCG is used in sequential combination with chemotherapy, fewer SSEs were reported. BCG induction with maintenance demonstrated a trend towards a lower frequency of SSEs during the maintenance regimen compared to the induction stage. Finally, our results indicate that low-dose BCG is less toxic than full-dose BCG through a comparison of the SSEs. The findings from this study allow for improved counselling of patients regarding expected side effects in accordance with their recommended treatment options for NMIBC.

## Supplementary Materials

The following supporting information can be downloaded at: https://www.mdpi.com/article/10.3390/cancers17020160/s1, Table S1: Induction and Maintenance Schedules of Included Studies.
