# Supplementary material for: Symptoms and Side Effects of Bacille Calmette–Guerin Therapy for Non-Muscle Invasive Bladder Cancer as Reported by Patients: A Systematic Review
Source: Cancers (Basel). 2025 Jan 7;17(2):160. doi: 10.3390/cancers17020160 (PMC11763679; doi:10.3390/cancers17020160)
Supplement: Supplementary file 1 [file cancers-17-00160-s001.zip › cancers-3319496-supplementary.pdf]

Table S1. Induction and Maintenance Schedules of Included Studies.

Author, Year	Therapy	Dosage	Induction Intervals	Maintenance Intervals
Ali El Dein, 2013	BCG* alternating with Epirubicin	120mg Pasteur strain for three weeks alternating with 50mg Epirubicin for three weeks	Weekly	Monthly epirubicin alternating with monthly BCG for one year
Agrawal, 2007	BCG at different doses	40mg	Weekly	Monthly for one year
Arends, 2016	BCG Tokyo	Either 80mg or 120mg	Weekly	
Addeo, 2010	Mitomycin	40mg/50mL saline	Weekly	Monthly for 10 months
	Gemcitabine	2000mg/50mL saline	Weekly	Monthly for 10 months
Burger, 2010	BCG Immucyst	19.2X10*8 CFU	Weekly	Three weekly at months three and six
Benha, 2015	BCG	90mg	Weekly	Three weekly at months three and six
Breyer, 2008	BCG Oncotice	1.6x10*8 CFU	Weekly	Three weekly at months three, six and twelve
Cheng, 2004	BCG Connaught	27mg/2.2-6.4x10*8 CFU/50mL saline for two hours	Weekly	
Decobert, 2008	BCG	120mg	Weekly	Three weekly
Di Stasi, 2006	BCG Connaught	BCG 120mg/50m saline	Weekly	Monthly
Eto, 1994	Epirubicin	30mg/30mL saline	Twice Weekly	Monthly
	Doxorubicin	30mg/30ml saline	Twice Weekly	Monthly
Friedrick, 2007	BCG RIVM strain and Mitomycin	2x10*8 CFU of RIVM for six weeks	Weekly	
Gruenwald, 1997	BCG Pasteur	120mg/50mL saline	Weekly	
Hendricksen, 2007	Epirubicin	50mg/50mL saline	Weekly	Monthly for five months
	Epirubicin	50mg/50mL saline	Weekly	Monthly for five months + one additional post TURBT
	Epirubicin	50mg/50mL saline	Weekly	Monthly for five months then further instillations at months nine and twelve
Hinotsu, 2010	BCG Connaught	81mg	Weekly	Three weeks at months three, six, nine, twelve, and eighteen
Koga, 2010	BCG	80mg	Weekly	

Krege, 1996	Mitomycin	Mitomycin 20mg/50mL saline	Second Weekly	Monthly
Kurg, 2012	BCG	81mg/40mL saline	Weekly	Two weeks at months three, six, nine, twelve, and eighteen twenty-four, thirty, thirty-six
Kuroda, 2004	Epirubicin	20mg/40mL saline	Weekly (x2), fortnightly (x7), monthly (x8)	
	Epirubicin	30mg/40mL saline	Weekly (x2), fortnightly (x7), monthly (x8)	
	Epirubicin	40mg/40mL saline	Weekly (x2), fortnightly (x7)	
Lori, 2002	BCG Tice	5x10*8 CFU /50ml saline	Weekly	Monthly
Mack, 1994	BCG Pasteur	75mg in 50mL water	Weekly	Monthly for eleven months
Martinez-Pineiro, 1989	Doxorubicin	50mg in 50mL water	Weekly	Monthly for eleven months
Martinez-Pineiro, 1995	BCG Connaught	81 mg	Weekly	Fortnightly for six instillations
Martinez-Pineiro, 2015	BCG	660-1920x10*6 CFU	Weekly	Once every three months for three years
Miguel, 2023	BCG OncoCITE	50mg/50mL saline	Weekly	Three weekly at three, six, twelve months
Morgia, 2002	BCG		Weekly	Three weeks at months three, six, nine, twelve, and eighteen twenty-four, thirty, thirty-six
Nour, 2015	BCG	45mg/50mL saline	Weekly	
Rigatti, 1990	BCG Pasteur	120 mg	Weekly	Monthly for one year then every three months for one year
Said, 2002	BCG	120mg/50mL saline	Weekly	Second weekly for three months then monthly for six months
Shuin, 1994	Doxorubicin	30mg/40mL saline	Second Weekly	
Si-Yang, 2010	Epirubicin	30mg/30ml saline	Weekly	Monthly
	Pirarubicin	30mg/30ml saline	Weekly	Monthly
	Hydroxycamptothecin	30mg/30ml saline	Weekly	Monthly
	Mitomycin	20mg/50mL distilled water	Weekly	Monthly
	Mitomycin	20mg for 6 weeks then monthly for three years	Weekly	Monthly
	Epirubicin	40mg/40mL saline	Weekly	Monthly for ten months

	Mitomycin	40mg	Weekly	Monthly for ten months
Wishahi, 1994	BCG	120mg	Weekly	Every three months for a year then once every six months for a year
Witjes, 1993	Mitomycin	30mg/50mL saline	Weekly	Monthly for six months
Yoo, 2012	BCG Tice	12.5mg	Weekly	Monthly for one year
